# Death and the maiden

**DOI:** 10.1002/jhm.70100

**Published:** 2025-06-11

**Authors:** Tina Arkee

**Affiliations:** ^1^ Vanderbilt University Medical Center Nashville Tennessee USA

It begins (or ends, depending on your perspective) with a farewell post on social media. There's a portrait of the artist from better days, their face blooming with health and radiance, accompanied by a message that can be distilled down to *I'm proud of what I have accomplished in my life and I'm not afraid to die*. They are holding a bouquet of flowers. I don't know enough about flower identification or floriography to tell if there is a message hidden in the bouquet. It seems like something they would do.

I feel like I know this person, but I don't, not really, and they certainly don't know me. We've exchanged messages over the years, but we were playing the roles of artist and eager patron rather than friends. All I know is what they've chosen to share with the world in the form of pixels and sound bites and semi‐confessionary writing, and yet I feel devastated. Their clinical trajectory took them from a diagnosis of breast cancer to a transition to comfort care in a little over 1 year. Fifteen months, to be precise.

I think this has hit me more than anticipated for two reasons. One, I formed a parasocial relationship with this person who shared their life and creations and magic online. Like thousands of others across the world, I felt lucky to exist in the same timeline as them; to bear witness to the beauty they created out of grief and trauma and an overwhelming zest for life. Two, it reminds me of some of the patients I've taken care of as an internal medicine resident, patients whose cases I grew invested in because walking into their rooms on rounds felt like looking into a mirror. These patients were all young and healthy until they weren't. They were young and healthy until malignant cells started growing in their bodies and, like seeds dispersed by the wind to take root in unchallenged territories, spread to distant sites. They were young and healthy until they died from complications of metastatic breast cancer. They were maidens who crossed paths with death far earlier than anticipated.

I felt connected to these patients despite only knowing them when they were running out of treatment options or too sick to be candidates for treatment, despite only catching glimpses of their personalities and lives outside of the hospital during our brief interactions. These connections made facilitating their transitions from treatment‐minded to comfort‐minded feel like I'd somehow failed them and their desires to live. These connections made opening their medical charts or coming across their social media accounts weeks or months later, only to learn of their deaths, feel like losing a dear friend.

I met most of these patients late in the courses of their diseases, often after they'd already been through weeks of toxic treatment intended to stop malignant cells in their path of destruction. There was a lot of existential angst on both sides—patient and physician—as we collectively grappled with the *unfairness* of their situations. There was a lot of frustration— frustration with a culture of medicine that assumes that young women's concerns are related to anxiety (the modern‐day hysteria) rather than a pathologic process because they're *too young* to have cancer, with intractable pain that keeps patients in the hospital instead of at their children's special events, with a healthcare system that makes it expensive to live *and* expensive to die. Some of these patients were diagnosed with high‐stage cancer despite feeling unwell for many months because they had difficulty physically accessing medical care, because they were too busy caring for their families. Others had physical access to medical care, but, uninsured and worried about the financial burden of treatment, attempted to self‐treat with herbal remedies that did little to stop the progression of an already aggressive disease. Still others made the difficult decision to transition to comfort care but encountered the unexpected barrier of being too clinically tenuous or financially insecure to die in the comfort of their own homes, and instead spent the last days of their lives in uncomfortable hospital beds.

I wish I knew more about *who* these patients were before their lives changed forever, before pathologies unintentionally became identities, when they were still people defined by their individual quirks and interests, rather than by the stage of their cancer and past treatments. I wish I had time to sit with them and their families and hear their stories about their hobbies, their jobs, and their travels. This was easier to accomplish as a medical student—I will never forget asking a young woman with widespread cancer about the meanings of her tattoos and exchanging pictures of our cats the day before she was discharged home after a prolonged hospitalization. As a resident, my ability to spend meaningful time with patients is limited by endless documentation, perpetually full hospitals necessitating as many early discharges as possible, and well‐meaning but poorly timed didactic sessions.

In the spring of my intern year, I took care of a young woman who, by no fault of herself or her oncologist, was dying from metastatic breast cancer. She was born into an unfortunate inheritance—she and nearly all of her female relatives carried a breast cancer gene mutation—and was thus betrayed by her body from a very young age. Multiple women in her family line had been affected by breast cancer, either personally or through a loved one; despite this, she was diagnosed at a more advanced stage due to difficulties accessing medical care, including lack of transportation and inability to take time off of work to attend appointments.

By the time that I met her, she was extremely sick—encephalopathic and weak because of brain metastases that required high‐dose steroids and whole brain radiation. She was also extremely young—exactly my age—and had a young child. I learned a little bit about her through her mother and sister's eyes, and spent some time with her family as they struggled with the critical decision of *what to do next*—wait to see if her clinical status would improve with continued steroids and radiation, with the hope of eventually trying another line of systemic treatment, or transition to comfort care and focus on quality time with family. They chose to transition to comfort care, and she had what I consider to be a good death—pain medications titrated to ward off pain and anxiety, loved ones at bedside to hold her hands as she transitioned to the other side of reality. I hope my friend, the artist, has a similar experience.

## CONFLICT OF INTEREST STATEMENT

The author declares no conflicts of interest.

